# Taraxasterol suppresses the proliferation and tumor growth of androgen-independent prostate cancer cells through the FGFR2-PI3K/AKT signaling pathway

**DOI:** 10.1038/s41598-023-40344-w

**Published:** 2023-08-11

**Authors:** Jinqiu Yang, Chulin Xin, Guangfen Yin, Juan Li

**Affiliations:** 1 School of Clinical Medicine, Dali University, Dali, 671013 Yunnan China; 2 School of Basic Medical Sciences, Dali University, 22 Wanhua Road, Dali, 671013 Yunnan China; 3 The First Affiliated Hospital of Dali University, Dali, 671013 Yunnan China

**Keywords:** Cancer, Cell biology, Diseases, Oncology

## Abstract

Prostate cancer (PCa) is prevalent among older men and difficult to survive after metastasis. It is urgent to find new drugs and treatments. Several studies show that taraxasterol (TAX) has important anti-inflammatory, anti-oxidative and anti-tumor effects. However, the function and mechanisms of TAX in PCa remain unclear. Here, we found that TAX could significantly suppress the viability and growth of androgen-independent PCa cells and down-regulate the expression of c-Myc and cyclin D1 in vitro. Mechanistically, PI3K/AKT signaling pathway was weakened and the expression of FGFR2 was reduced after TAX treatment in androgen-independent PCa cells. Moreover, TAX evidently inhibited the tumor growth in nude mice and the expression of c-Myc, cyclin D1, p-AKT and FGFR2 were down-regulated in xenograft tumor. These results indicate that TAX suppresses the proliferation of androgen-independent PCa cells via inhibiting the activation of PI3K/AKT signaling pathway and the expression of FGFR2, which means TAX may be a novel anti-tumor agent for later PCa treatment.

## Introduction

Prostate cancer (PCa) is one of the common malignancies in men, ranking second in morbidity and fifth in mortality. Despite advances in screening and diagnosis, the incidence of PCa continues to increase by years^1^. The occurrence and development of PCa is a very complicated process, and there are many possibilities in the pathogenesis. Genetic and epigenetic changes of multiple genes, including fusion of TMPRSS2 with ERG^2^, amplification or overexpression of Myc^3,4^, deletion or inactivation of PTEN and TP53^5,6^, mutation or amplification of the androgen receptor (AR), initiate and promote the occurrence of PCa^7–9^. Inflammation, oxidative stress, abnormal telomerase activity and shortening of telomere length, cell senescence and abnormal expression of non-coding RNA all promote the process of PCa^10,11^. PCa is highly treatable in its early stages. The treatment of localized PCa includes active surveillance, local radiotherapy and prostatectomy^12^. Androgen deprivation therapy (ADT) by surgical or chemical castration is the most commonly used and effective treatment for PCa patients. Unfortunately, PCa patients often develop resistance to ADT, and cancer cells will undergo genetic changes and metastasize to distant tissues and organs, developing into castration-resistant prostate cancer (CRPC)^13^. The 5-year survival rate of PCa patients diagnosed with local lesions as high as 99%, but it’s only 30% for patients with metastatic PCa^14^. The complex pathogenesis and the sharp decline in survival rate make it urgent to find new strategies or targets for the prevention and treatment of PCa.

Dysregulation of phosphatidylinositol 3 kinase (PI3K)/protein kinase B (AKT) is closely associated with the development and progression of human cancer. Previous researches have shown that the activation of PI3K/AKT signaling pathway and alterations of oncogenic components in the PI3K/AKT signaling pathway can promote tumorigenesis by regulating cell migration, proliferation, survival and angiogenesis^15,16^. In addition, many studies have indicated that the PI3K/AKT signaling pathway is associated with the development of PCa. PI3K/AKT signaling pathway is regulated by the ErbB, EGFR and HER families to promote PCa cell growth. Inflammatory factors (CCR9, IL-6 and TLR3) can modulate the PCa cell apoptosis via PI3K/AKT signaling. The PTEN/PI3K/AKT pathway and PI3K/AKT/mTOR signaling pathway can accommodate PCa cell metastasis and invasion^17^. Moreover, PTEN deletion activates the PI3K/AKT/mTOR pathway, which is the most common molecular mechanism of CRPC and one of the causes of ADT resistance^18^. Fibroblast growth factor receptor 2 (FGFR2), has often happened somatic hotspot mutations, structural amplification and fusion in multiple cancers^19^. FGFR2 is mainly responsible for transduction of FGF signals into PI3K-AKT signaling casccascals^20^. A previous study found that the loss of FGFR2 is related to the malignant progression of prostate cancer and it will be a clinical therapeutic target^21^.

Chemotherapy can obviously improve the prognosis and survival of PCa patients, but the adverse reactions are serious and prone to drug resistance^22^. Therefore, it is urgent to find new anti-tumor drugs to maintain the life of PCa patients. Scientific studies have shown that many natural products and extracts could be used as the potential anti-tumor agents of PCa in recent years^23^. Taraxasterol (TAX), (3β, 18α, 18α)-Urs-20(30)-en-3-ol, is a pentacyclic-triterpene compound with various biological activities. TAX can be extracted from many types of plants and is one of the main active components of Taraxacum officinale Wigg^24^. Previous studies have proved that TAX has anti-inflammatory, anti-oxidative and anti-carcinogenic properties^25^. TAX can inhibit tumor cells growth of many types of cancer including nasopharyngeal carcinoma, breast carcinoma, colon carcinoma, cervix carcinoma, ovary carcinoma and so on^26–28^. TAX significantly suppresses the proliferation and tumor formation of gastric cancer cells by inhibiting EGFR/AKT1 signaling pathway^29^. TAX also can inhibit RNF31/p53 axis-driven cell proliferation in colorectal cancer by targeting RNF31^30^. In addition, TAX regulates the expression of Bax, Bcl-2 and cyclin D1 by up-regulating Hint1 transcription, selectively inhibits the proliferation of hepatocellular carcinoma cells, and induces G0/G1 cell cycle arrest and apoptosis^31^. However, the effect of TAX on the growth of PCa in vitro and in vivo and its mechanism have not yet been revealed.

Our study is aimed at investigating whether TAX can inhibit the proliferation of PCa cells and which signaling pathway and genes closely related to the anti-tumor effect of TAX. Here, we demonstrated that TAX can significantly suppress the proliferation of androgen-independent PCa cells and down-regulate the expression of c-Myc and cyclin D1 in vitro. In addition, we also found that TAX inhibited activation of the PI3K/AKT signaling pathway and reduced the expression of FGFR2 in androgen-independent PCa cells. Finally, we verified TAX evidently inhibited the tumor growth in nude mice and decreased the expression of c-Myc, cyclin D1, p-AKT and FGFR2 in xenograft tumor. These results show that TAX suppresses the proliferation of androgen-independent PCa cells through regulating the PI3K/AKT signaling pathway and FGFR2. Based on this, TAX could be a potential agent for the later clinical treatment of PCa.

## Results

### TAX suppresses the proliferation of androgen-independent PCa cells

**Figure 1:**
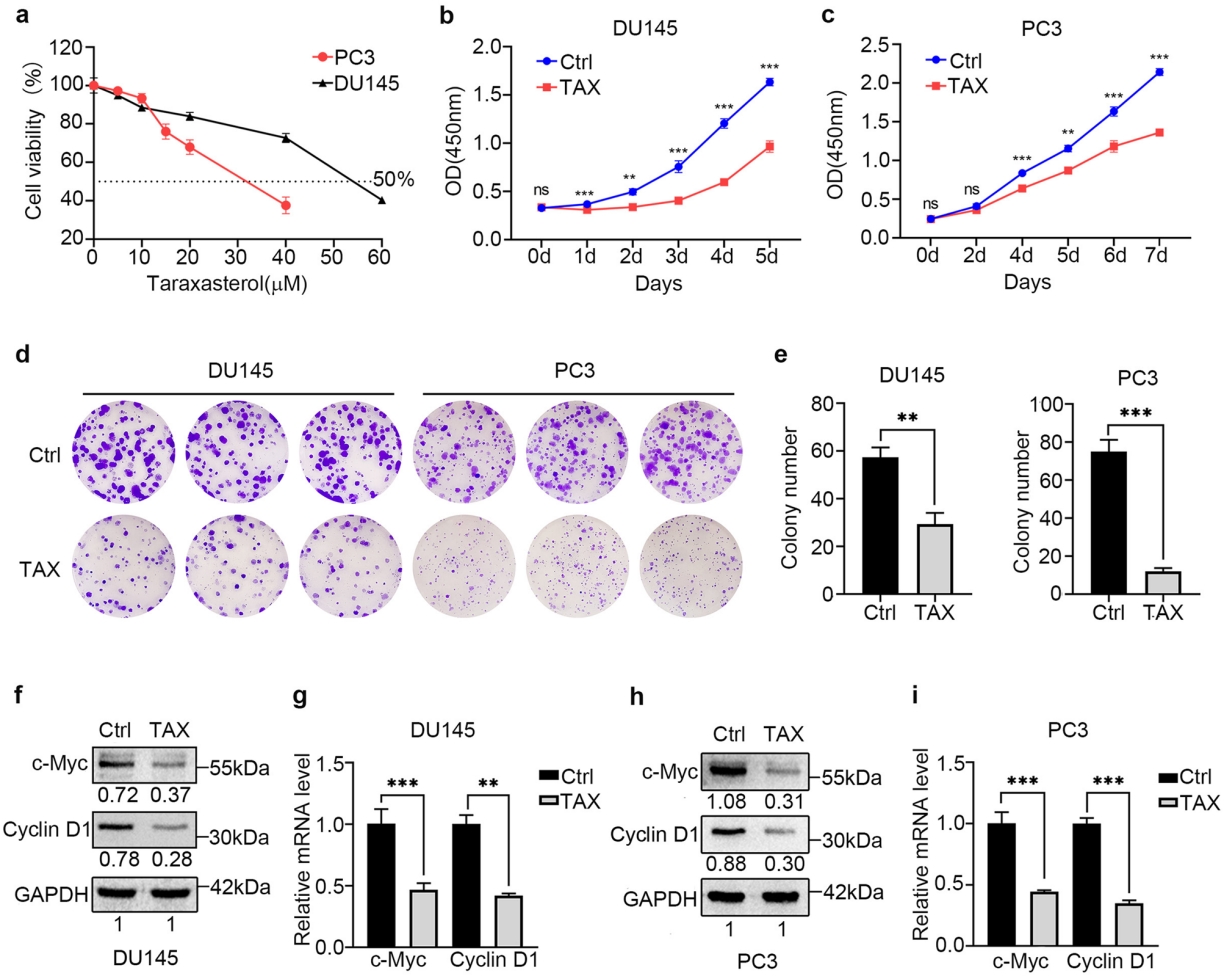
TAX inhibited the proliferation of PCa cells. (**a**) TAX reduced the cell viability of PCa in a dose-dependent manner. The DU145 and PC3 cells were treated with TAX (0, 5, 10, 20, 40 and 60 μM for DU145 cells; 0, 5, 10, 15, 20 and 40 μM for PC3 cells) for 48 h and the viability in individual groups of cells was determined by CCK-8. (**b**, **c**) The proliferation of DU145 and PC3 cells treated by TAX at the IC_50_ dose (56 µM for DU145 cells; 30 μM for PC3 cells) for 0–7 d was detected by CCK-8. (**d**, **e**) The colony formation ability of DU145 and PC3 cells were detected by crystal violet staining after treated with TAX for two weeks. (**f**–**i**) The expression of c-Myc and cyclin D1 in DU145 and PC3 cells after treated with TAX was examined by western blotting and real-time qPCR. The blots of c-Myc, cyclin D1 and GAPDH were cropped from different parts of the same gel. *ns* not significant; *p < 0.05; **p < 0.01; ***p < 0.001.

To investigate the optimal treatment concentration of TAX in different androgen-independent PCa cells, DU145 and PC3 cells were treated with varying concentrations of TAX for 48 h to determine the viability of individual groups of cells. The results of CCK-8 assay showed that TAX reduced the viability of androgen-independent PCa cells in a dose-dependent manner and the 50% inhibition concentrations (IC_50_s) of TAX were 56 µM for DU145 cells and 30 μM for PC3 cells (Fig. 1a). Compared with the control cells without TAX treatment, the growth of DU145 and PC3 cells treated with TAX at the IC_50_ dose was significantly inhibited (Fig. 1b, c). Moreover, according to the colony formation assay, the treatment of TAX markedly reduced the colony size and number of DU145 (The size of colony ≥ 1.0 mm^2^) and PC3 (The size of colony ≥ 0.5 mm^2^) cells (Fig. 1d, e). In addition, the expression of c-Myc and cyclin D1 decreased after TAX treatment at both protein and mRNA levels in DU145 and PC3 cells (Fig. 1f–i). These data demonstrated that the TAX could inhibit the proliferation of androgen-independent PCa cells.

### TAX attenuates the PI3K/AKT signaling pathway and down-regulates the expression of FGFR2

**Figure 2:**
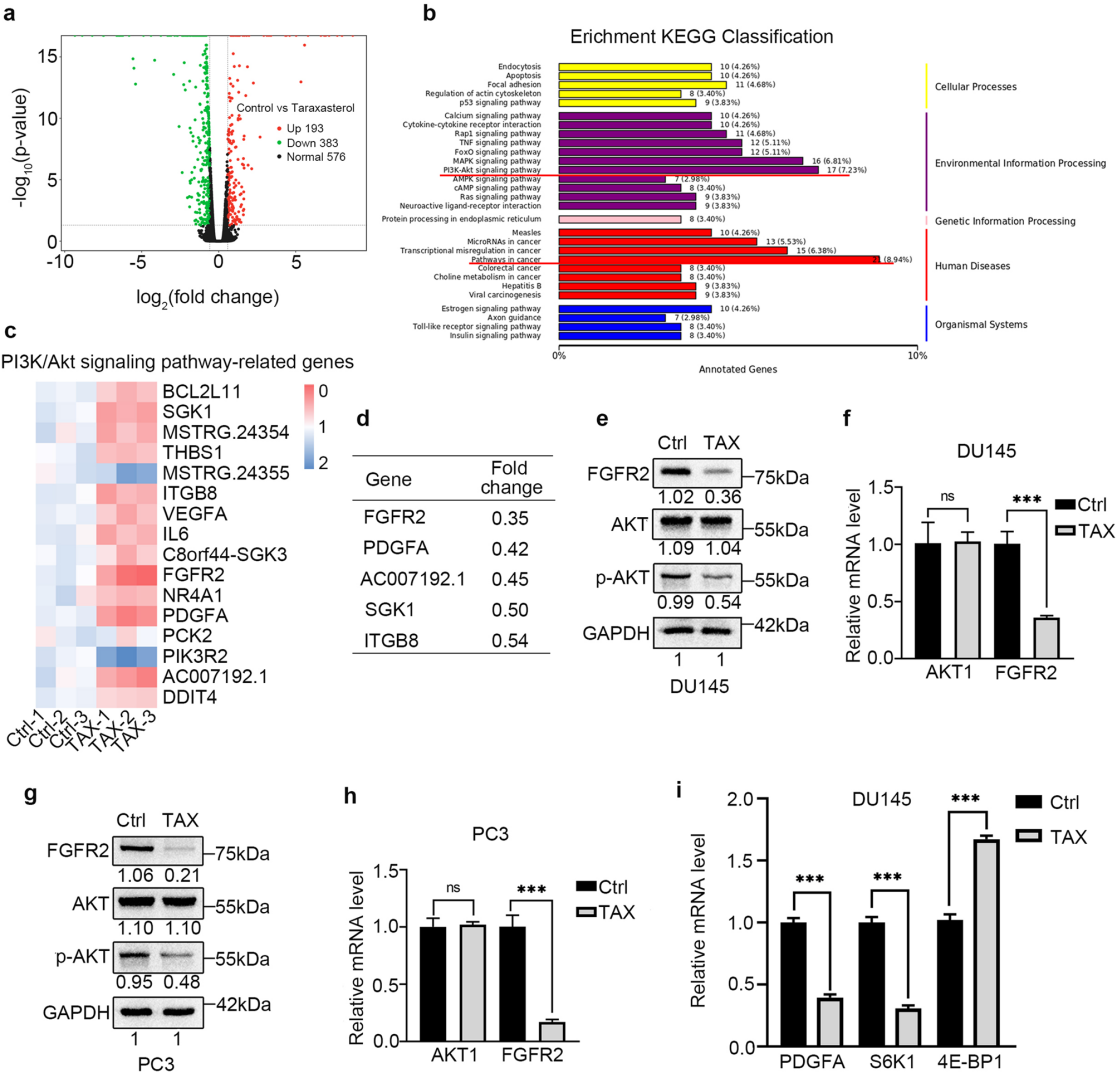
TAX attenuated the FGFR2-PI3K/AKT signaling pathway in PCa cells. (**a**) The Volcano Plot showed the number of the altered genes after treated with TAX for 48 h in DU145 cells. (**b**) The KEGG pathway analysis^43–45^ revealed the changes of pathways after treated with TAX. (**c**-**d**) The Heatmaps showed the 16 PI3K/AKT signaling pathway-related differentially expressed genes after treated with TAX and the most significant five genes of them. (**e**–**h**) The expression of FGFR2, AKT and p-AKT in DU145 and PC3 cells after treated with TAX for 48 h was examined by western blotting and real-time qPCR. The blots of FGFR2, AKT, p-AKT and GAPDH were cropped from different parts of the same gel. (**i**) The expression of PDGFA, 4E-BP1, S6K1 in DU145 cells after treated with TAX for 48 h was examined by real-time qPCR. *ns* not significant; *p < 0.05; **p < 0.01; ***p < 0.001.

Next, the mechanism by which TAX suppressed the proliferation of androgen-independent PCa cells was explored. According to the RNA sequencing in DU145 cells treated with or without TAX, in comparison with control cells, 193 genes were up-regulated and 383 genes were down-regulated by TAX (Fig. 2a). KEGG pathway analysis revealed the most obvious changes of pathways after treated with TAX were cancer-related pathways, among which the change of the PI3K/AKT signaling pathway was the most obvious (Fig. 2b). Differential expression gene analysis showed that there were 16 PI3K/AKT signaling pathway-related differentially expressed genes after treated with TAX (Fig. 2c), while FGFR2 was the most significant one of them (Fig. 2d). Based on these findings, the expression of AKT, p-AKT and FGFR2 at the protein and mRNA level after treated with TAX was verified in DU145 and PC3 cells. The results of western blotting and real-time qPCR demonstrated that TAX reduced the phosphorylation of AKT and down-regulated the expression of FGFR2 in androgen-independent PCa cells (Fig. 2e–h). Moreover, we also examined the mRNA levels of PDGFA and two classical downstream genes (4E-BP1, S6K1) of mTOR and Akt in the PI3K/Akt pathway in DU145 cells. The results showed that the TAX inhibited the expression of PDGFA and S6K1, and upregulated the expression of 4E-BP1 to varying degrees at the mRNA levels in DU145 cells (Fig. 2i).

### The inhibitory effect of TAX on androgen-independent PCa cells could be reversed by Recilisib

**Figure 3:**
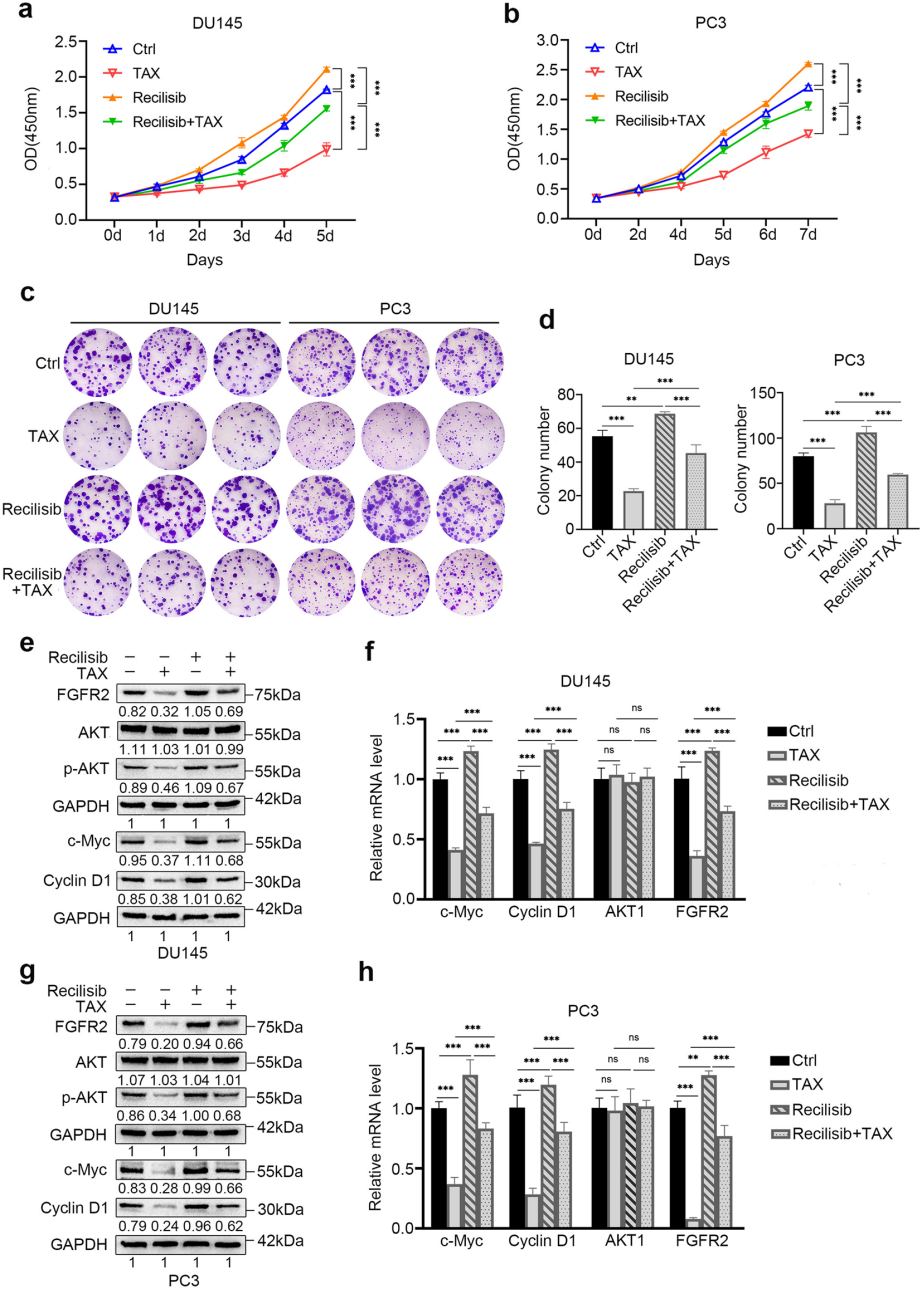
Recilisib reversed TAX's inhibitory effect on PCa cells. (**a**, **b**) The proliferation of DU145 and PC3 cells treated with individual or combined TAX (56 μM for DU145 cells; 30 μM for PC3 cells) and Recilisib (80 µM/L) for 0–7 d was detected by CCK-8. (**c**, **d**) The colony formation ability of DU145 and PC3 cells were detected by crystal violet staining after treated with individual or combined TAX and Recilisib for two weeks. (**e**, **h**) The expression of c-Myc, cyclin D1, AKT, p-AKT and FGFR2 in DU145 and PC3 cells after treated with individual or combined TAX and Recilisib was examined by western blotting and real-time qPCR. The blots of c-Myc, cyclin D1 and GAPDH were cropped from different parts of the same gel and the blots of FGFR2, AKT, p-AKT and GAPDH were cropped from different parts of another same gel. *ns* not significant; *p < 0.05; **p < 0.01; ***p < 0.001.

Recilisib is a radioprotectant, which can activate the activities of AKT and PI3K in cells^32^. DU145 and PC3 cells were treated with individual or combined TAX and Recilisib to investigate whether the Recilisib could reverse the inhibitory effect of TAX on androgen-independent PCa cells. The cell growth curve showed that Recilisib could enhance the proliferation of DU145 and PC3 cells and partially abolish the affection of TAX(Fig. 3a, b). Colony formation assay suggested that the co-treatment of TAX and Recilisib increased the colony size and number of DU145 and PC3 cells compared to the cells of individual TAX treatment (Fig. 3c, d). The results of western blotting and real-time qPCR demonstrated that the expression of c-Myc, cyclin D1, p-AKT and FGFR2 of DU145 and PC3 cells could be up-regulated after Recilisib treatment compared to the cells of individual TAX treatment at protein and mRNA level (Fig. 3e–h). These results support that Recilisib could partially reverse the anti-proliferation effect of TAX on DU145 and PC3 cells, demonstrating that TAX inhibits the proliferation of androgen-independent PCa cells via weakening PI3K/AKT signaling pathway and reducing the expression of FGFR2.

### TAX inhibits the tumor growth of androgen-independent PCa cells in vivo

**Figure 4:**
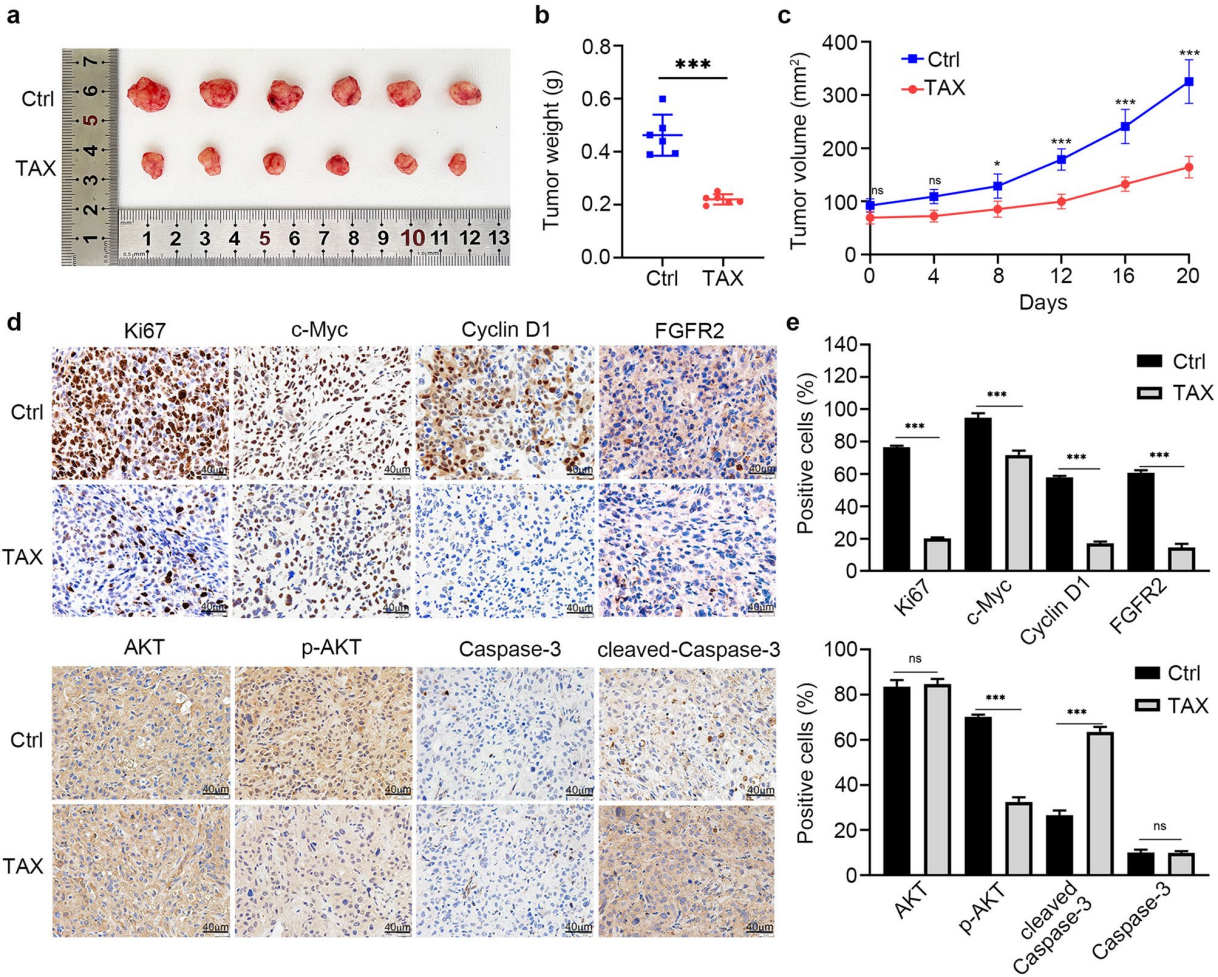
TAX inhibited the tumor growth of DU145 cells in vivo. (**a**–**c**) The tumor morphology, size, volumes and weights of DU145 cells after treated with TAX for 21 d in BALB/c nude mice. (**d**–**e**) Immunochemistry staining of Ki67, c-Myc, cyclin D1, AKT, p-AKT, FGFR2, Caspase-3 and cleaved-Caspase-3 in xenograft tumors of DU145 cells. Scale bars, 40 µm. *ns* not significant; *p < 0.05; **p < 0.01; ***p < 0.001.

To verify the above results, a subcutaneous DU145 cells xenograft model in BALB/c nude mice was established to further evaluate the anti-tumor effect of TAX in vivo. DU145 cells were inoculated into male BALB/c nude mice which were immunosuppressed, with or without TAX treatment for 21 days. Compared with the control group, TAX significantly reduced the tumor growth of DU145 cells indicated by tumor images, tumor weights and tumor volumes (Fig. 4a–c). In the tumor xenografts, immunohistochemistry staining demonstrated that TAX treatment decreased the number of Ki67-positive cells and reduced the expression of c-Myc, cyclin D1, p-AKT and FGFR2 (Fig. 4d, e). These results in vivo indicates that the tumor growth of PCa cells can be inhibited by TAX. In addition, we detected the expression of apoptotic related Caspase-3 and cleaved-Caspase-3 in PCa xenograft of DU145. We found that the TAX increased the expression of cleaved-Caspase-3. These results showed that after TAX treatment the apoptosis of androgen-independent PCa increased (Fig. 4d, e).

## Discussion

Although the implementation of prostate-specific antigen (PSA) testing could early detect PCa effectively^33^, the mortality of PCa especially metastatic PCa, remains grim now^1,14^. ADT is the most effective initial treatment for metastatic PCa, but almost all patients will eventually progress to CRPC^34^. Patients with CRPC are often resistant to chemotherapeutic agents and the prognosis is poor. To find more effective therapeutic agents of PCa is a major clinical challenge. Many natural products are widely used in clinical treatment based on their remarkable anti-tumor effect on various cancers and less cytotoxic than other chemotherapeutic agents^35^. Our study investigated the inhibition effect of TAX on the proliferation and tumor growth of androgen-independent PCa cells.

TAX could be isolated from many types of plants including Taraxacum officinale Wigg^24,25^. In recent years, the anti-tumor function of TAX has gained increased attention. Previous researches demonstrated that TAX could suppress the tumor cells growth in many types of cancer, such as breast carcinoma, colon carcinoma, cervix carcinoma, ovary carcinoma, and gastric cancer via multiple signaling pathway, including EGFR/AKT1^29^ and PI3K/AKT signaling pathway^36^ to attenuate the progression of cancer^26–29^. However, there are few studies on the effect of TAX in PCa cells. A study showed that TAX can significantly inhibit the proliferation of PC3 cells^37^, but the mechanism has not yet been revealed. Here, our study showed that TAX could inhibit the proliferation and colony formation of PC3 and DU145 cells, down-regulating the expression of c-Myc and cyclin D1 (Fig. 1). The inhibition of the tumor growth of androgen-independent PCa cells in vivo further proved the anti-proliferation effect of TAX. These results highlight the potential therapeutic value of TAX in the later treatment of PCa.

A lot of evidence demonstrate that PI3K/AKT signaling pathway is abnormal activation in many types of cancer to promote tumorigenesis by regulating nutrient metabolism, cell proliferation, survival, migration, and angiogenesis^16^. Therefore, PI3K/AKT signaling pathway potential inhibitors may be critical for effective treatment of cancers in the clinic. Fibroblast growth factor receptors 2 (FGFR2) is expressed in a variety of cancers, such as oral mucosal, esophageal, gastric, colorectal, pancreatic, pulmonary, breast, endometrial, cervical and prostate cancers, to promote the carcinogenesis and cancer progression, thus it has been considered as a new target for cancer treatment^38^. Moreover, it has been proved that FGFR2 was overexpressed in the PCa epithelial cells associated with poor differentiation^39^. Here, we found that TAX could prominently suppress PI3K/AKT signaling pathway and reduce the expression of FGFR2 in androgen-independent PCa (Fig. 2). This result was also confirmed in vivo by tumor formation of androgen-independent PCa cells in nude mice (Fig. 4). In addition, compared to the cells of individual TAX treatment, the co-treatment of TAX and Recilisib (an agonist of PI3K/AKT signaling) increased the proliferation and colony formation of androgen-independent PCa cells, and up-regulated the expression of c-Myc, cyclin D1, p-AKT and FGFR2 (Fig. 3), which means that the re-activation of PI3K/AKT could partially abolish the anti-proliferation effect of TAX on androgen-independent PCa cells. Our study suggests that a possible mechanism by which TAX represses the proliferation and tumor growth of androgen-independent PCa cells through down-regulating PI3K/AKT signaling pathway and the expression of FGFR2. These results provide new evidence of TAX as a new candidate agent for the later treatment of PCa, giving new support for FGFR2 as a therapeutic target for PCa.

This study only studied the mechanism of TAX inhibits the proliferation of DU145 and PC3 PCa cells. In fact, the two PCa cell lines PC3 and DU145 used in our study were derived from patients after bone and brain metastases and representative of the type-I androgen depletion independent PCa in which AR is not expressed^40,41^. The study of the inhibitory effect and mechanism of TAX on PC3 and DU145 PCa cells is also of great significance for the later treatment of PCa. Moreover, we had tested the effects of TAX in AR-dependent cells lines LNCaP and C4-2B. The results of CCK-8 assay showed that TAX reduced the viability of LNCaP and C4-2B cells, and the expression of c-Myc and cyclin D1 decreased after TAX treatment at both protein and mRNA levels. At the same time, we also found that TAX reduced AR expression in LNCaP and C4-2B cells, while AR signaling is crucial for the proliferation of androgen-dependent prostate cancer cell. Looking at the literature, AR signaling and PI3K-AKT signaling have cross-dialogue, which jointly regulate the proliferation of androgen-dependent prostate cancer cells^15,16,42^. Therefore, for androgen-dependent prostate cancer cells, the mechanism that TAX inhibits their proliferation is more complex. So, we think the mechanism that TAX inhibits the proliferation of androgen-dependent and androgen-independent prostate cancer cells is not the same. This is a very interesting study, we are carrying out experimental work on it.

In summary, our study demonstrated that TAX suppressed the proliferation and tumor growth of androgen-independent PCa cells in vitro and in vivo. Mechanically, TAX could inhibit the PI3K/AKT signaling pathway and reduce the expression of FGFR2 to play the anti-proliferation role in androgen-independent PCa cells. Moreover, our results showed that some other signaling pathways were also affected by TAX. It’s interested in further exploring the other effects of TAX on PCa cells and changes of specific signaling pathways and targets in PCa cells under the effect of TAX in future studies. Hopefully, there will be more and more studies about TAX in PCa cells and giving more understanding of the molecular mechanisms of TAX, which may help TAX become a new choice of clinical treatment agent of PCa.

## Materials and methods

All experimental protocols were approved by Dali University. All methods were performed in accordance with relevant guidelines and regulations.

### Reagents and chemicals

MEM medium, RPMI-1640 medium, fetal bovine serum (FBS), phosphate-buffered saline (PBS), and penicillin–streptomycin (Pen Strep) were purchased from Biological Industries (Israel). CCK-8 reagent was purchased from Proteintech (Wuhan, China). Dimethyl sulfoxide (DMSO), 4% paraformaldehyde, and 0.1% crystal violet were purchased from Solarbio (Beijing, China). TAX (purity ≥ 98%) was purchased from ChemFaces (Wuhan, China). AKT activator (Recilisib) was purchased from MCE (New Jersey, USA).

### Cell culture and treatment

Human PCa cell lines DU145 and PC3 were gifted from Professor JD Dong, South University of Science and Technology of China (Shenzhen, China). DU145 cells were cultured in MEM medium and PC3 cells were cultured in RPMI-1640 medium supplemented with 10% FBS and 1% Pen Strep at 37 °C under a humidity incubator of 5% CO_2_. TAX was dissolved in DMSO and diluted in medium according to the experimental concentration.

### Cell viability assay

Cell viability was performed by the CCK-8 assay. 5 × 10^3^ DU145 cells /well or 1 × 10^4^ PC3 cells/well were seeded in 96-well plates. After 12 h, DU145 and PC3 cells were treated with TAX and Recilisib in combination or alone for 0–7 d, then added CCK-8 reagent to each well with each time point and determined the absorption at 450 nm after 2 h.

### Colony formation assay

200 DU145 cells/well or 500 PC3 cells/well were seeded into 6-well plates and treated with TAX and Recilisib in combination or alone for 2 weeks. Then the colonies were fixed with 4% paraformaldehyde and stained by 0.1% crystal violet. After photographed, the number of colonies were counted by using Image J software.

### Real-time qPCR assay

Total RNAs were isolated from cells using FastPure Cell/Tissue Total RNA Isolation Kit (Vazyme, Nanjing, China), and 2 μg total RNA were reverse-transcribed using the HiScript III 1st Strand cDNA Synthesis Kit (+ gDNA wiper) (Vazyme). Real-time qPCR was performed with the ChamQ Universal SYBR qPCR Master Mix (Vazyme) using the StepOnePlus™ real time PCR system (Eppendorf, Hamburg, Germany). Primer sequences used are listed in Supplementary Table S1.

### Western blot analysis (WB)

Total protein of DU145 and PC3 cells was extracted with 2× loading buffer (Solarbio, Beijing, China). Antibodies used in western blotting are shown below: GAPDH (1:50000, 60004-1-Ig, Proteintech), c-Myc (1:1000, ab32072, Abcam), cyclin D1 (1:20000, ab134175, Abcam), Akt (1:2000, 4691, CST), p-Akt (1:2000, 4060, CST), FGFR2 (1:500, ab289968, Abcam), HRP-conjugated Affinipure Goat Anti-Rabbit IgG(H + L) and Peroxidase-conjugated Affinipure Goat Anti-Mouse IgG(H + L) (1:5000, SA00001-2 and SA00001-1, Proteintech). The blots were quantified by Image J software. Uncropped scans can be found in Figure S1.

### RNA sequencing (RNA-Seq)

To define the gene expression changes of DU145 cells after TAX (56 µM) treatment for 48 h, total RNAs were isolated using Trizol reagent (Invitrogen, Carlsbad, CA). The high-throughput RNA sequencing was conducted by Tsingke Biotechnology Co., Ltd. (Beijing, China). Genes with a fold change (FC) ≥ 1 and p value < 0.05 between control and TAX-treated groups were identified as significant differentially expressed genes (DEGs).

### Xenograft mice studies

Male BALB/c nude mice (4-week-old) were purchased from Beijing HFK Bioscience Co., Ltd. DU145 cells (2 × 10^6^) in 100 µL PBS were injected into the flanks of nude mice. After three weeks, mice were randomly divided into control (normal saline) or TAX (10 mg/kg) groups (n = 6/group), which were given by gavage every day. Tumor volume was measured every 4 days and calculated with the formula of V = (length × width × width)/2. After 21 days, mice were euthanized by injecting an overdose of anesthetic (1% pentobarbital sodium) and tumors were surgically dissected, weighed and fixed in 4% paraformaldehyde. Animal studies were approved by Ethics Review Committee of Dali University (ethical committee approval no.2021-PZ-018) and were conducted in accordance with the ARRIVE guidelines.

### Immunohistochemistry (IHC)

4% Paraformaldehyde-fixed tissues were embedded in paraffin and cut into 4 μm thick section. After deparaffinized, rehydrated, repaired antigen, treated with 3% H_2_O_2_ and blocked with 10% normal goat serum, tissue sections were incubated with primary antibodies (Ki67, c-Myc, cyclin D1, AKT, p-AKT, FGFR2) at 4 °C overnight. Next day tissue sections were incubated with PolymerHRP secondary antibodies (MXB, Fuzhou, China) to perform the chromogenic reaction using DAB (MXB) and dye nuclei with hematoxylin (MXB). Finally, tissue sections were mounted and photographed. The staining positive rate were analyzed with ImageJ software.

### Statistical analysis

All experiments were repeated at least three times. All numerical results data were presented as mean ± SD and performed in Graph Pad Prism version 5.0 software using T test. *p* < 0.05 was considered statistically significant.

### Ethics approval and consent to participate

This study was accepted by the Ethics Review Committee of Dali University.

### Supplementary Information

Supplementary Information.

## Data Availability

The datasets generated and analyzed during the current study are available in the GEO repository (accession number: GSE222413, token number: crqvkwwgrnubfqz).

## Abbreviations

PCa: Prostate cancer
TAX: Taraxasterol
PI3K: Phosphatidylinositol-3-kinase
AKT: Protein kinase B
FGFR2: Fibroblast growth factor receptor 2
