# Supplementary material for: Taraxasterol suppresses the proliferation and tumor growth of androgen-independent prostate cancer cells through the FGFR2-PI3K/AKT signaling pathway
Source: Sci Rep. 2023 Aug 11;13:13072. doi: 10.1038/s41598-023-40344-w (PMC10421874; doi:10.1038/s41598-023-40344-w)
Supplement: Supplementary file 1 — Supplementary Information. [file 41598_2023_40344_MOESM1_ESM.docx]

**Fig.S1: Unprocessed blot images for western blotting results.**

**
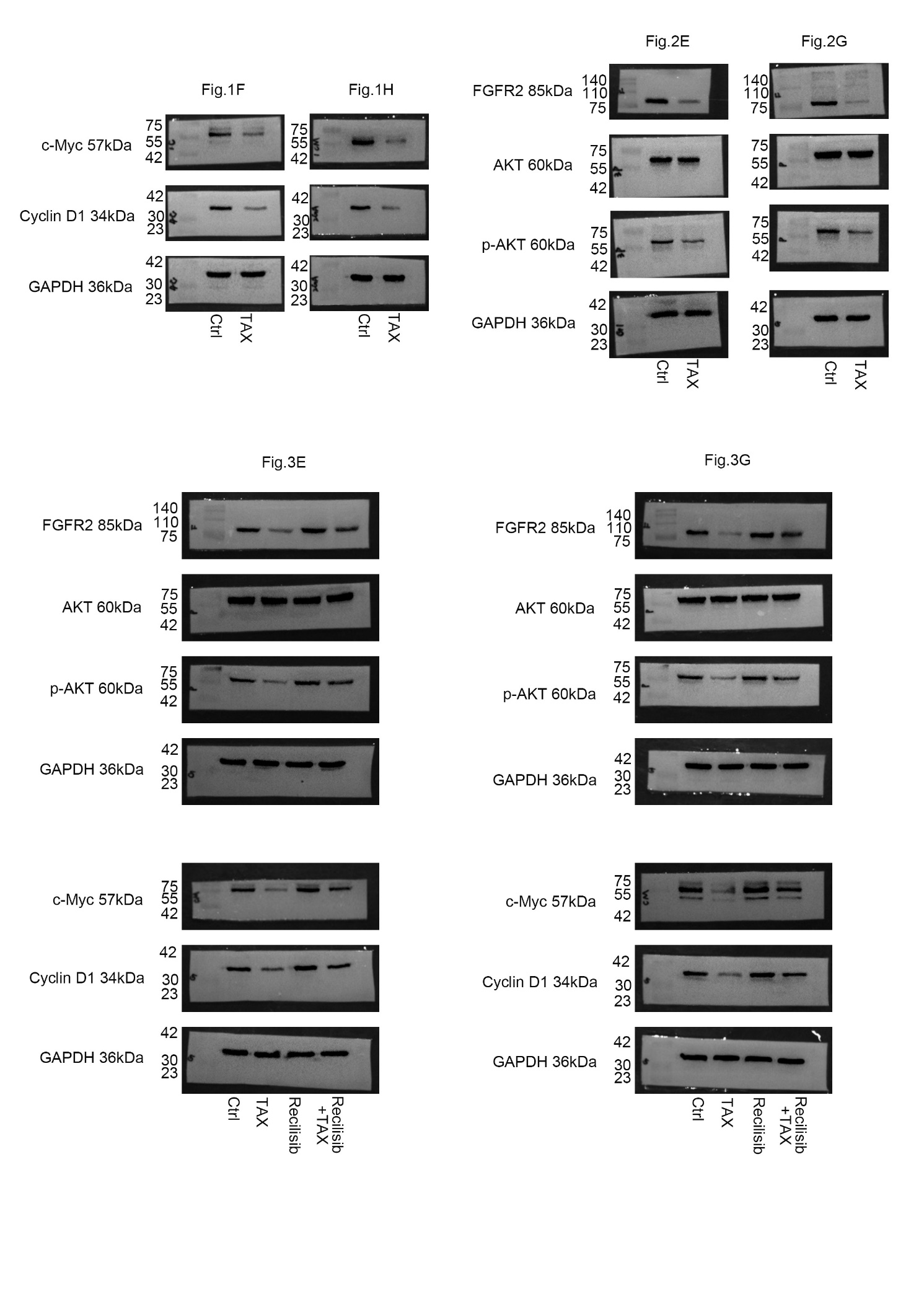
**

**Table S1: Primer sequences used in various PCRs in the study.**

| Gene | Primer (5’ to 3’) |
| --- | --- |
| GAPDH | Forward: GGAAGCTTGTCATCAATGGAAATC |
|  | Reverse: TGATGACCCTTTTGGCTCCC |
|  |  |
| FGFR2 | Forward: AGAGTGATGTCTGGTCCTTCGG |
|  | Reverse: TGGCTGGCTTATCCATTCTGTG |
|  |  |
| AKT1 | Forward: TACTCTTTCCAGACCCACGACC |
|  | Reverse: CCCGGTACACCACGTTCTTCT |
|  |  |
| Cyclin D1 | Forward: AGCTGTGCATCTACACCGAC |
|  | Reverse: GAAATCGTGCGGGGTCATTG |
|  |  |
| c-Myc | Forward: GGTAGTGGAAAACCAGCAGCC |
|  | Reverse: CTCCTCGTCGCAGTAGAAATACG |
|  |  |
| PDGFA | Forward: AGTGTCAAGTGCCAGCCCTC |
|  | Reverse: CGGATTCAGGCTTGTGGTCG |
|  |  |
| S6K1 | Forward: TCCGATCACCTCGAAGATTTATTG |
|  | Reverse: CATCTGCTCTATGCCACTTGTTTC |
|  |  |
| 4E-BP1 | Forward: CTATGACCGGAAATTCCTGATGG |
|  | Reverse: CCCGCTTATCTTCTGGGCTA |
